# ^99m^Tc-labeled monosaccharide kits: development methods and quality control

**DOI:** 10.1038/s41598-020-61707-7

**Published:** 2020-03-20

**Authors:** Elena Stasyuk, Viktor Sкuridin, Alexander Rogov, Roman Zelchan, Vladimir Sadkin, Natalya Varlamova, Evgeny Nestеrov

**Affiliations:** 1 Tomsk Polytechnic University, 30 Lenina Avenue, 634050 Tomsk, Russia; 2 Cancer Research Institute, Tomsk National Research Medical Center, Russian Academy of Sciences, 5 Kooperativny street, 634050 Tomsk, Russia

**Keywords:** Cancer imaging, Cancer imaging, Drug development, Drug development

## Abstract

The paper presents the procedure for planning an experiment to create standard sets of reagents for a technetium-99m generator based on glucose derivatives. All stages are presented from researching the required quantities of a substance, a reducing agent, a stabilizer and auxiliary components to developing lyophilized kits and conducting quality control. The radiochemical purity of radiopharmaceuticals prepared on the basis of the developed kits ranged from 90.0 to 99.0%. We also showed the functional suitability of the developed preparations on C57B1/6j mice with an implanted malignant tumor - Lewis lung carcinoma.

## Introduction

Glucose derivatives are potent scaffolds for the development of radiopharmaceuticals for cancer diagnosis as they target one of the key metabolic changes exhibited by tumor cells - an increased level of glucose metabolism^1–7^. The design and synthesis of diagnostic radiopharmaceuticals (RPs) based on monosaccharides is covered by several research groups. The majority of them employ the common strategy involving drug preparation by mixing the reagents immediately prior to the injection. This approach lacks the quality control of prepared RP and is difficult to implement in medical laboratories. The most convenient monosaccharide formulation lyophilized powder. It has an extended shelf life, can be delivered to the clinics for the subsequent preparation of RP via mixing with the radionuclide. However, such formulations for single-photon emission computed tomography are not avaliable to date.

**Figure 1:**
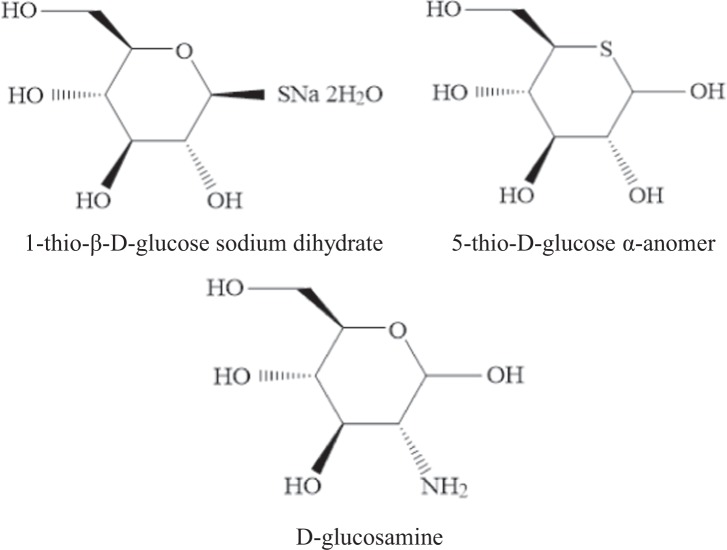
Structural formulas of monosaccharides under study.

Earlier, aiming to develop novel technetium-99m labeled glucose-based RPs, we investigated monosaccharides containing sulfur and nitrogen as they are capable of forming complexes with technetium-99m^8^. The following compounds were studied: 1-thio-D-glucose, 5-thio-D-glucose and D-glucosamine, as well as their salts and hydrates (Fig. 1). Herein, our goal was to study the process of labeling these substances with the technetium-99m radionuclide. As a result, for the first time we propose an experimental method for the design of lyophilized kits based on glucose derivatives and a protocol for their radioactive labeling.

## Experimental

### Chemicals

All the reagents were purchased from Sigma-Aldrich ACS grade and used without further purification. Technetium-99m was obtained from chromatographic ^99^Mo/^99m^Tc generator «^99m^Tc-GT-TOM» produced by Tomsk Polytechnic university (TPU) - Tomsk, Russia.

### ^99m^Tc labeling

#### 5-thio-β-D-glucose Labeling

Preparation of 5-thio-β-D-glucose substance lyophilizates was carried out as follows: 1 mL of a solution containing 15 mg of the glucose derivative, 0.175 mg of tin chloride and 0.5 mg of ascorbic acid were placed in a 10 mL vial. The vials without prior freezing were applied to a freeze-dryer at a pressure of 0.2 Pa and a condenser temperature of −50 °C for at least 24 h, including 4 h of drying in the upper chamber. For the radiolabeling 4 mL of sodium pertechnetate solution [^99m^Tc] with an activity of 1.0 GBq was added to the obtained lyophilized samples and incubated at room temperature for 30 min. Radiochemical purity (RCP) control and the formation of the complex were monitored by TLC.

#### 1-thio-β-D-glucose labeling

Preparation of 1-thio-β-D-glucose substance lyophilizates was carried out as follows: 1 mL of a solution containing 2.5 mg of the glucose derivative, 0.25 mg of tin chloride and 0.5 mg of ascorbic acid were placed in a 10 ml vial. The vials without prior freezing were applied to a freeze-dryer at a pressure of 0.2 Pa and a condenser temperature of −50 °C for at least 24 h, including 4 h of drying in the upper chamber. For the radiolabeling 4 mL of sodium pertechnetate solution [^99m^Tc] with an activity of 1.0 GBq was added to the obtained lyophilized samples and incubated at room temperature for 30 min. Radiochemical purity (RCP) control and the formation of the complex were monitored by TLC.

#### D-glucosamine labeling

Preparation of lyophilizates of D-glucosamine substances was carried out as follows: 1 mL of a solution containing 20 mg of the glucose derivative, 0.140 mg of tin chloride and 200 μl of hydrochloric acid with a concentration of 0.05 mole was placed in a 10 mL vial. The vials without prior freezing were applied to a freeze-dryer at a pressure of 0.2 Pa and a condenser temperature of −50 °C for at least 24 h, including 4 h of drying in the upper chamber. For the radiolabeling 4 mL of sodium pertechnetate solution [^99m^Tc] with an activity of 1.0 GBq was added to the obtained lyophilized samples and incubated at room temperature for 30 min. Radiochemical purity (RCP) control and the formation of the complex were monitored by TLC.

### High performance liquid chromatography

#### Metod 1

A chromatographic method for the lyophilized drug based on D-glucosamine was developed using a ZORBAX SB column (C18, 4.6×150 mm, 5 μm, Agilent, USA). The following gradient elution with 0.1% trifluoroacetic acid water solution as “A” and 0.1% trifluoroacetic acid in acetonitrile as “B” was used at a flow rate of 1 mL/min: 0–3 min A:B = 100:0; 3–6 min A:B = 50:50; 6–10 min A:B = 0:100. Analysis was performed by UV absorption (λ = 254 nm).

#### Metod 2

A chromatographic method for the lyophilized drug based on 5-thio-β-glucose and 1-thio-β-glucose was performed using a ZORBAX NH2column (4.6×250 mm, 5 μm, Agilent, USA). The following isocratic elution (A: B = 30: 70 A: water; B: acetonitrile) during 20 min, was used at a flow rate of 1 mL/min. Analysis was performed by refractometric detection.

#### TLC

Instant thin-layer chromatography on silica gel (TLC-SG, Sorbfil, Russia) was performed using different mobile phases. Acetone was used to determine the amount of free ^99m^TcO4 - (*Rf* = 0.9*–*1.0)- impurity *A*, and а mixture of C_2_H_5_OH: NH_4_OH: H_2_O = 2:5:5 for ^99m^Tc colloid (*Rf* = 0.0)- impurity *В*. Strips were analyzed by gamma counter. Radiochemical purity was calculated as follows: RCP = 100- (*A* + *B*),%

#### Stability of ^99m^Tc-labeled derivatives glucose

The *in vitro* stability of radioactively labeled derivatives glucose in an aqueous solution was determined by adding a 1000-fold molar excess of cysteine in PBS and incubating at room temperature followed by monitoring the radiochemical purity after 1, 2, 3 and 6 h by TLC. Reference solutions were the same solutions of labeled glucose derivatives, but instead of an excess of cysteine solution, the same volume of PBS solution was added. Both sample groups were analyzed by TLC.

### Biodistribution

Biodistribution of novel radiopharmaceutical formulations was studied *in vivo* on Lewis lung carcinoma model in mice. The animals were treated according to the rules of the European Convention for the Protection of Vertebrate Animals (Strasbourg, 1986). The experimental protocols were approved by Cancer Research Institute Biomedical Ethics Committee, Protocol number 14. All invasive manipulations with animals were performed using inhalation or drug anesthesia.

Cell line of Lewis lung carcinoma (Bank of Cell Lines of the Russian N. Blokhin Russian Cancer Research Center, Russian Academy of Medical Sciences, Moscow) was passaged in C57B1/6j mice. Each mouse was given an injection in the leg muscle with a suspension containing 1–3×10^6^ viable tumor cells. During this stage of the study, 40 mice with the weight of 30–35 g were included in the experiment. The average volume of the tumor node, measured instrumentally, was 2.3 ± 0.7 cm^3^.

The radiopharmaceutical was administered to mice intravenously at 0.1 mL, with a volume activity of 200 MBq/mL. At certain time intervals after the injection (15, 40, and 120 min), static scintigraphy of the whole body of animals was performed on an E.CAM Signature 1800 gamma camera (Siemens, Germany). The data was processed using the specialized software package Esoft 5.5 (Siemens, Germany). A differential photo-peak discriminator was set up at 140 keV, with a window width of 20%, using a parallel low-energy high-resolution collimator. At the time of the study, the animals were placed horizontally to the surface of the gamma-camera detector so that the entire body of the animal appeared in the field of view. To assess the intensity of the inclusion, the percentage of the inclusion of the radiopharmaceutical was calculated in relation to the symmetric area and the entire body of the animal.

## Results and discussion

### Study of the solubility of the main component

Before conducting experimental studies on the development of a new radiopharmaceutical, it is necessary to investigate the solubility of its main component in various media: water for injection, 0.9% NaCl solution (physiological saline), hydrochloric acid, alkaline solution, dehydrated C_2_H_5_OH and other supposed media planned to be used in the experiments.

The temperature regime in this case should be in the range of 15 to 25 °C, and the pH value in the range of 7 ± 3. The study of the solubility of substances in various media is also necessary for selecting the composition of chromatographic mixtures for subsequent radiochromatography of the produced RP and estimation of their radiochemical yield and radiochemical purity.

As an example, Table 1 presents the results of the study of the solubility of all of the above glucose derivatives.

**Table 1:** Testing of the solubility of glucose derivatives in various solvents, n = 3.

№	Solvent	Solubility, рН D- glucosamine	Solubility, рН 5 - thio-D-glucose	Solubility, рН 1- thio-D-glucose
1	C_2_H_5_OH	Insoluble, 7	Slightly soluble, 7	Insoluble, 8
2	0.5 M NaOH solution	Slightly soluble, 12	Soluble, 12	Soluble, 12
3	0.5 M HCl solution	Soluble, 2	Soluble, 2	Soluble, 2
4	0.9% NaCl solution	Soluble, 5	Soluble, 5	Soluble, 9
5	H_2_O	Soluble, 5	Soluble, 5	Soluble, 10
6	C_2_H_5_OH: NH_4_OH: H_2_O ÷ 2:5:5	Soluble, 5	Soluble, 5	Soluble, 9
7	Acetone	Soluble, 5	Soluble, 5	Soluble, 10

The data of the table show that all the derivatives are readily soluble in water and physiological saline, which makes it possible to use these media for preparing the drug.

It should also be noted that 1-thio-D-glucose derivative in media 4–7 has a higher pH value. The solubility of all derivatives in acetone allows the determination of unreduced ^99m^TcO_4_^-^ monosaccharides in labeling products by thin layer chromatography (TLC). Similarly, in a mixture of C_2_H_5_OH: NH_4_OH: H_2_O it is possible to determine the impurity content of hydrolyzed technetium oxide (^99m^ТсО_2_) using TLC. In this mixture, labeled glucose derivatives and non-associated ^99m^TcO_4_^-^ pertechnetate ions rise with the mobile-phase front, and hydrolyzed technetium remains on the start line.

#### The choice of a reducing agent for ^99m^TcO_4_^−^ pertechnetate ions

The seven-valent ^99m^Tc, present in the eluate, isolated from the technetium-99m generator as sodium pertechnetate solution (Na^99m^TcO_4_), does not have complexing properties. Therefore, for its interaction with chelating agents its reduction is carried in advance

Several different reducing agents can be used to reduce pertechnetate, ^99m^Tc: Sn (II) tin ions, ascorbic acid in combination with Fe (II), sodium borohydride, sodium thiosulfate, hydrazine, sulfhydryl and aldehyde compounds. Most often, divalent Sn (II) tin in the form of dichloride dihydrate SnCl_2_ ∙ 2Н_2_О is used for its reduction in the preparation of lyophilizates. In order to identify its maximum allowable content in the reaction mixture, preliminary studies are conducted to determine the amount of reducing agent in which the admixture of unreduced 99mTcO4- into the drug does not exceed 5% of its initial activity.

#### Development of qualitative and quantitative composition of drugs

The main requirement for radiopharmaceuticals is the stability of the chemical bond of the original substance with the radioactive marker. This is often achieved by introducing various auxiliary substances into the composition of the reaction mixture, for example, additives stabilizing the valence state of the reacting substances, especially Sn (II). For glucose derivatives, ascorbic acid is a stabilizing effective additive.

To identify the necessary and sufficient quantities of reducing and stabilizing agents in the mixtures of basic substances, a wide range of their concentrations was previously studied. The obtained optimal component ratios providing the best yields of the labeled product and its radiochemical purity are given in Table 2.

**Table 2:** Content of basic and auxiliary substances in the reaction mixtures for the preparation of drugs based on glucose derivatives.

Substance	Content, mg		
	RP “5-thio-D-glucose, ^99m^Тс“	RP “1-thio-D-glucose, ^99m^Тс“	RP “D- glucosamine, ^99m^Тс“
Glucose Derivative	15.0 mg (5-thio-D-glucose α-anomer)	2.5 mg (1-thio-β-D-glucose sodium dihydrate)	20.0 mg (D-glucosamine)
Tin chloride dihydrate	0.175 mg	0.21 mg	0.140 mg
Ascorbic acid	0.5 mg	0.5 mg	—
Solution of HCl (C = 0.05М)	—	—	200 µl
Radiochemical purity (RCP) n = 5	95.0 ± 0.5%	99.0 ± 0.3%	90.0 ± 0.5%
Radiolabeling Yield (RLY) n = 5	<70.0%	<93.0%	<32.0%

From the results presented in the table should be emphasized that the reaction mixture for the preparation of D-glucosamine-based radiopharmaceutical preserves the stability of the valence state of Sn (II) in the presence of HCl, at that the yield of radiolabeling was the lowest among the glucose derivatives studied by us and was 32.0% with a radiochemical purity of 90.0 ± 0.5%. The output of radiolabeling for 5-thio-D-glucose was more than 70.0% with a radiochemical purity of 95.0 ± 0.5%, for a 1-thio-D-glucose preparation, the output of radiolabeling yield (RLY) was the highest and was more than 93.0% with a radiochemical purity of 99.0 ± 0.3%.

The stability of the complexes of 5-thio-D-glucose and 1-thio-D-glucose with technetium was quite high for 4 hours, which is confirmed by the data of RCP. The radiochemical purity of the 5-thio-D-glucose complex of the initial was 96.21% after an hour was 96.15%, after 2 hours 95.91% and 95.51%, 94.51% after 3 and 4 hours, respectively.

The radiochemical purity of the 1-thio-D-glucose complex initially was 99.34%. After 1 hour, RCP was 99.25%, after 2 hours it was 99.15% and 99.0%, and 98.7% after 3 and 4 hours, respectively.

The stability of the D-glucosamine complexes with technetium was slightly lower compared to previous glucose derivatives. The initial RCP value of90.1% decreased to 89.7% in an hour and was reduced to 89.1%, 88.7% and 75.2% after 2, 3 and 4 hours respectively.

#### Development of the procedure for obtaining a lyophilized reagent for the preparation of radiopharmaceutical

When elaborating the conditions for obtaining lyophilized reagents for the preparation of monosaccharides labeled with technetium-99m with a long shelf life, it is necessary to study the influence of the lyophilization process on the qualitative characteristicsof the drugs.

Based on the results of the studies carried out, several programmes should be tested when choosing the type of lyophilisate preparation. At the same time, parameters such as average temperature, time of primary and final drying, holding (or absence) of pre-freezing can be varied.

All the test mixtures of reagents should be prepared in a small volume of ~ 1 ml to ensure maximum drying conditions. The depth of drying was controlled by the gravimetric method, the preparation was dried until reaching constant weight in vials.

After final drying, the flasks with a mixture of reagents are folded with aluminum caps. Then a necessary volume of technetium-99m eluate is injected through the plug using a syringe, the resulting preparation is mixed and/or incubated, followed by sampling for chromatogramming. It is possible that technological tests of the lyophilisates obtained sometimes require additional correction of the previously developed reagent composition. The parameters of the process of lyophilization of reagents for the preparation of radiopharmaceutical based on glucose derivatives are presented in the Table 3.

**Table 3:** Parameters of the lyophilization process of reagents kits for the preparation of radiopharmaceutical based on glucose derivatives.

Name of the parameter	RP “5-thio-D-glucose, ^99m^Тс”	RP “1-thio-D-glucose, ^99m^Тс”	RP «“D- glucosamine, ^99m^Тс”
Volume of mixture in 1 flask	Not more than 1 ml	Not more than 1 ml	Not more than 1 ml
Pre-freezing	no	2 h (−50 °С)	2 h (−50 °С)
Vacuum level of lyophilization	0.2 Pa	0.2 Pa	0.2 Pa
Lyophilization temperature	−50 °С	−50 °С	−50 °С
Time of lyophilization, h	24	22	21
Temperature in the upper drying chamber	+10 °С + 15 °С	+10 °С + 15 °С	+10 °С + 15 °С
Drying time in the upper lyophilic chamber, h	4.0	5.0	5.5

#### Development of the technological scheme for the preparation of radiopharmaceutical

**Figure 2:**
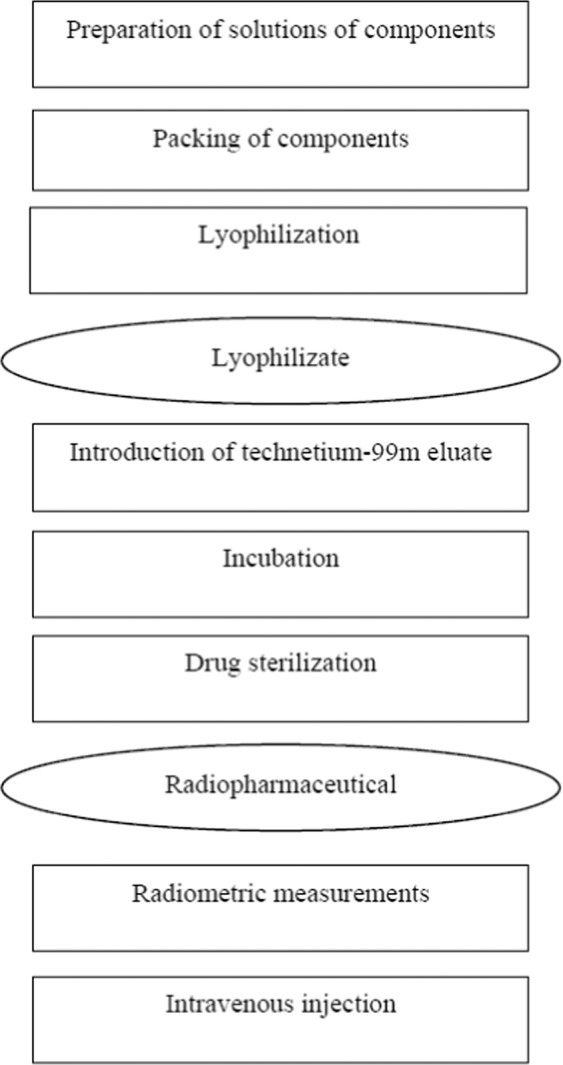
Technological scheme of radiopharmaceutical preparation based on technetium-99m labeied derivative of glucose.

Proceeding from the preliminary development of the optimal composition of the drug and conditions for lyophilization of the mixture of reagents for its preparation, for the development of radiopharmaceutical samples to monitor their quality and study the functional suitability, it is necessary to develop a complete technological scheme (Fig. 2) in which the sterilization process of the drug must be taken into account.

#### Choice of the methods for analytical quality control of kit and radiopharmaceutical

According to current requirements, quality control of radiopharmaceuticals prepared from standard reagent kits (lyophilizates) by mixing them with a radionuclide-containing drug for labeling should include tests for the authenticity of the used substance and all other reagents that make up lyophilizate, as well as tests for pH, volumetric activity, radiochemical purity, bacterial endotoxin content and sterility of the introduced radiopharmaceutical. Practically all listed parameters are determined by standard procedures given, for example, in the State Pharmacopoeia of the Russian Federation, XIII ed., Volumes 1, 2 and 3 (SP XIII)^9^. At the same time, analytical methods for the determination of the components that form freeze-drying agents containing monosaccharides in combination with reducing agents and other excipients require careful consideration, taking into account the possible mutual influence of these components on the results of their determination.

Several methods exist for the determination of glucose derivatives: photometric^10^, spectral (Nuclear magnetic resonance spectroscopy^11^ and Infrared spectroscopy^12^), chromatographic^13–15^, and others. Due to the low volatility and good solubility of mono- and disaccharides in water, the methods of liquid chromatography and, in particular, high-performance liquid chromatography (HPLC) are most frequently used for their determination^16^ and for semiquantitative determination - thin-layer chromatography^17^.

In our experiments, a high-pressure liquid chromatograph “Agilent 1200” with a refractometric and UV detectors and two types of columns ZORBAX NH2 and ZORBAX SB-C18 using different elution modes.

**Figure 3:**
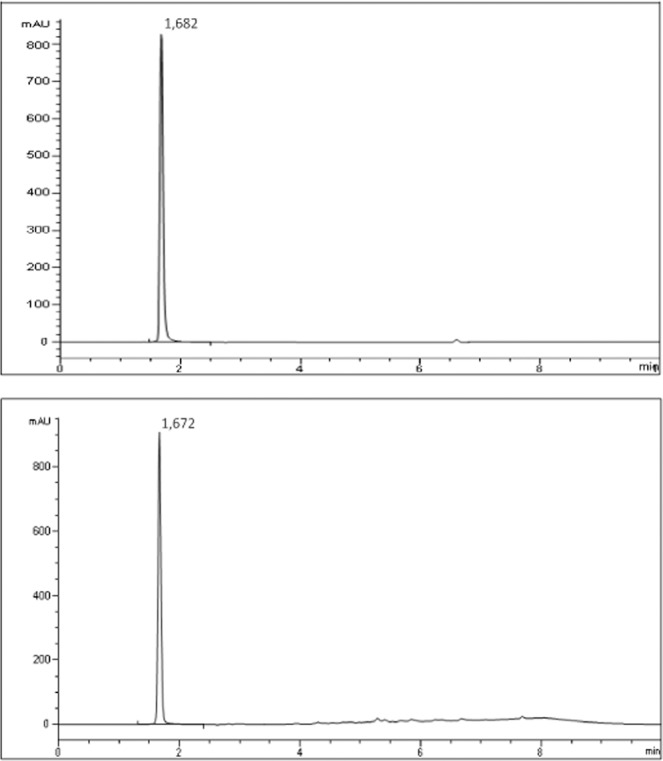
HPLC chromatogram determination of the content of D-glucosamine in the reagent for the preparation of radiopharmaceuticals using method 1, Etalon and the reagent based on D-glucosamine for the preparation of radiopharmaceuticals, respectively.

As shown by our research method 1 using a UV detector, ZORBAX SB-C18 columns and gradient elution mode cannot be applied to preparations based on 5-thio-D-glucose and 1-thio-D-glucose, i.e. ascorbic acid, which in this mode of determination is not separated from the main substance and significantly increases the height of the peak of the main substance 1TG and 5TG. Using method 1, the content of D-glucosamine in the reagent for the preparation of RFP was determined (Fig. 3).

**Figure 4:**
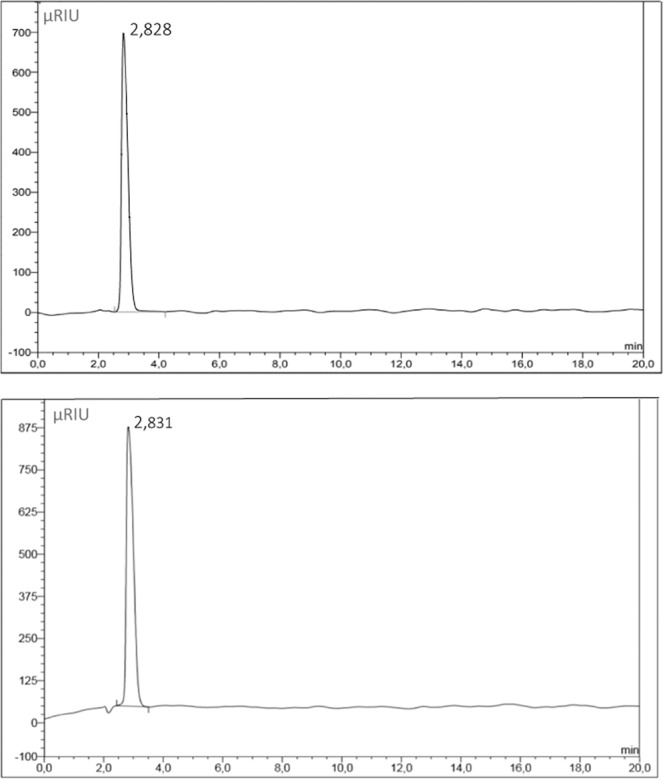
HPLC chromatogram determination of the content of 1-thio-D-glucose in the reagent for the preparation of radiopharmaceuticals using method 2, Etalon and the reagent based on 1-thio-D-glucose for the preparation of radiopharmaceuticals, respectively.

Therefore, a second determination method was developed to determine the amount of the substance 5-Thio-D-glucose and 1-Thio-D-glucose in the lyophilisates obtained. Using a refractometric detector, ZORBAX NH2 column and isocratic elution mode. As our studies have shown, this method allowed us to exclude the contribution of ascorbic acid in determining the amount of 5-thio-D-glucose and 1-thio-D-glucose (Fig. 4).

The device is convenient in that the results of the analysis are displayed in the form of chromatograms and tables in which found peaks, their area and the retention time are noted.

To carry out qualitative and quantitative determination of Sn (II) ions, a procedure based on the reaction of Sn (II) interaction with potassium perrhenate in the acidic medium in the presence of potassium thiocyanate was used. According to this procedure, the determination of tin was carried out on a “UNICO” spectrophotometer with a maximum absorption at a wavelength of 353 ± 2 nm in the range from 340 to 370 nm using standard calibration curves.

When developing methods for the determination of tin in the solution of reagents for the production of glucose-based radiopharmaceuticals, the influence on the definition of related components such as 1-thio-D-glucose, 5-thio-D-glucose, D-glucosamine and ascorbic acid was investigated (Table 4).

**Table 4:** Results of experimental determination of the concentration of tin chloride by the spectrophotometric method in the presence of various components.

The composition of the analyzed mixture	Found С_Sn(II),_ mg/ml	Calculated С_SnСl2·2H2O,_ mg/bottle	Average value С_SnСl2·2H2O,_ mg/bottle, (n = 4)
30 μl − 7 mg/ml SnCl_2_ (0,210 mg) + 3,970 ml 0.9% NaCl. Total volume reagent 4 ml.	0.0263	0.200	0.201 ± 0.007
	0.0268	0.204	
	0.0276	0.210	
	0.0250	0.190	
30 μl −7 mg/ml SnCl_2_ (0,210 mg) +50 μl АК (10 mg/ml) + 1 ml 1-thio-D-glucose (2.5 mg) + 2.920 ml 0.9% NaCl. Total volume reagent 4 ml.	0.0250	0.190	0.201 ± 0.007
	0.0263	0.200	
	0.0276	0.210	
	0.0270	0.205	
30 μl −7 mg/ml SnCl_2_ (0,210 mg) + 1 ml 1-thio-D-glucose (2.5 mg) + 2.970 ml 0.9% NaCl. Total volume reagent 4 ml.	0.0250	0.198	0.201 ± 0.005
	0.0263	0.200	
	0.0257	0.195	
	0.0276	0.210	
30 μl −7 mg/ml SnCl_2_ (0,210 mg)+ 50 μl АК (10 mg/ml) + 3.920 ml 0.9% NaCl. Total volume reagent 4 ml.	0.0270	0.205	0.202 ± 0.005
	0.0262	0.199	
	0.0257	0.195	
	0.0275	0.209	

As our studies of mixtures of different compositions showed, the presence of concomitant components in the analyzed solution (1-thio-D-glucose, 5-thio-D-glucose, D-glucosamine, ascorbic acid) does not interfere with the exact determination of tin (II) ions by this method.

#### Evaluation of the functional suitability of the preparation by determining its pharmacokinetic characteristics

After the development of the technology of preparation of lyophilizates and methods of quality control of the drug, the study of its pharmacokinetic characteristics, the laws of its accumulation and distribution in the body, tropism to the tumor tissue, the dynamics of excretion is carried out, the coefficient of differential accumulation (CDN) is determined. All these characteristics allow to conduct a full evaluation of the functional suitability of radiopharmaceutical.

**Figure 5:**
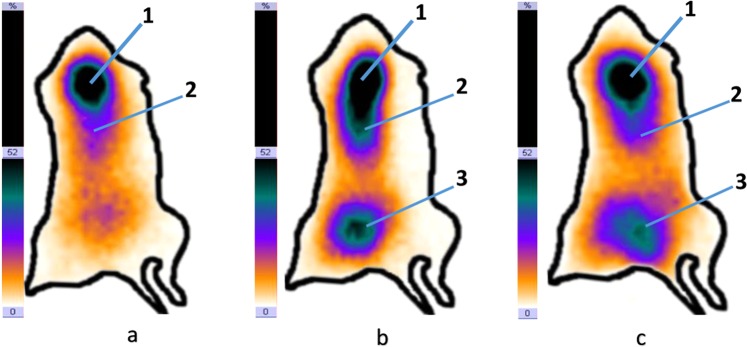
Scintigram of radiopharmaceutical “1-thio-D-glucose, ^99m^Tc” in the body of the experimental animal 15(a), 40(b), 120(c) minutes after drug administration: 1- place of injections, 2 - thoracic organs, 3 - tumor LLC.

As an example, Fig. 5 shows a gamma scintigram of a mouse with implanted malignant tumor - Lewis lung carcinoma implanted into the right thigh (Bank of Cell Lines, N.N. Blokhin Cancer Research Center, the Russian Academy of Sciences, Moscow). The experiment was carried out in accordance with the provisions of the European Convention for the Protection of Vertebrates used for experiments or for other scientific purposes (Strasbourg, March 18, 1986),^18^ in mice of the C57BL/6 line with Lewis carcinoma (LLC). The studies were carried out by the method of planar scintigraphy using the E.CAM Signature 1800 gamma camera (Siemens, Germany) with a low-energy high-resolution collimator.

On the scintigram (Fig. 5a) 15 minutes after the administration of 20 MBq of the preparation, the injection site (infraorbital sinus) and the organs of the chest and abdominal cavity are visualized. The infraorbital sinus was chosen as the site of injection farthest from the transplanted tumor, given the small size of the experimental animal. The introduction of the drug into the tail vein of an animal was difficult due to the toxic effects of the implanted tumor. 40 minutes after the administration of the drug (Fig. 5b), the injection site (infraorbital sinus), the organs of the thoracic cavity and the implanted tumor of the right thigh are visualized. 120 minutes after the drug administration (Fig. 5c), an implanted tumor of the right thigh is visualized, aswell as a decreased intensity of the drug inclusion in the tumor is noted. Moreover, the injection site (infraorbital sinus), the organs of the chest cavity arevisualized. For better visualization of the tumor, experimental animals emptied the bladder.

## Conclusion

As a result of the studies, the technological instruction for the creation of technetium-99m labeled derivatives of glucose was developed and proposed. The main stages of the production of lyophilized reagent kits based on monosaccharides were considered and formulated in the course of performing research to study the process of labeling glucose derivatives with a technetium-99m radionuclide at Tomsk Polytechnic University in the framework of the federal program on the topic: “Preclinical studies of radiopharmaceutical on the basis of ^99m^Tc labeled glucose derivative for radionuclide diagnostics of oncological diseases” №2015-14-N08-0008 of the Ministry of Education and Science of the Russian Federation. At present, preclinical trials of radiopharmaceutical based on 1-thio-β-D-glucose have been performed in full together with the Tomsk Scientific Research Institute of Oncology of Tomsk Scientific and Technical Center of the Russian Academy of Sciences for the purpose of their subsequent use for single-photon emission computed tomography of oncological diseases.
